# Copper in the colorectal cancer microenvironment: pioneering a new era of cuproptosis-based therapy

**DOI:** 10.3389/fonc.2024.1522919

**Published:** 2025-01-09

**Authors:** Qixuan Feng, Yue Sun, Zhe Yang, Zhiyu Wang, Zhangyi Chen, Fang Liu, Lingxiang Liu

**Affiliations:** ^1^ The First School of Clinical Medicine, Nanjing Medical University, Nanjing, Jiangsu, China; ^2^ Department of Oncology, The First Affiliated Hospital of Nanjing Medical University, Nanjing, Jiangsu, China; ^3^ The Second School of Clinical Medicine, Nanjing Medical University, Nanjing, Jiangsu, China; ^4^ School of Basic Medical Sciences, Nanjing Medical University, Nanjing, Jiangsu, China

**Keywords:** cuproptosis, CRC, cuproplasia, copper homeostasis, tumor microenvironment

## Abstract

Copper, an essential trace element and biochemical cofactor in humans plays a critical role in maintaining health. Recent studies have identified a significant association between copper levels and the progression and metastasis of cancer. Copper is primarily absorbed in the intestinal tract, often leading to an imbalance of copper ions in the body. Colorectal cancer (CRC), the most common cancer originating in the intestines, thrives in an environment with elevated copper concentrations. Current research is focused on uncovering the relationship between copper and CRC which has introduced new concepts such as cuproplasia and cuproptosis, significantly deepening our understanding of copper’s influence on cell proliferation and death. Cuproplasia is a kind of cell proliferation mediated by the co-regulatory activities of enzymes and non-enzymatic factors, while cuproptosis refers to cell death induced by excessive copper, which results in abnormal oligomerization of lipacylated proteins and the reduction of iron-sulfur cluster proteins. Exploring cuproplasia and cuproptosis opens new avenues for treating CRC. This review aims to summarize the critical role of copper in promoting colorectal cancer, the dual effects of copper in the tumor microenvironment (TME), and strategies for leveraging this unique microenvironment to induce cuproptosis in colorectal cancer. Understanding the relationship between copper and CRC holds promise for establishing a theoretical foundation for innovative therapeutic strategies in CRC.

## 1 Introduction

Colorectal cancer (CRC) is the most common primary bowel cancer, and its incidence is increasing. Currently, it is the third most common cause of cancer and the second leading cause of cancer-related death worldwide, with an incidence and mortality rate of 10.7% and 8.1%, respectively, in 2023 (1). CRC is characterized by multistage progression, often associated with a poor prognosis, and is frequently diagnosed at an advanced stage (2). The development of sustained and highly effective drugs for CRC is challenging due to the drug-resistance of CRC (3). The tumor microenvironment (TME), which is the direct environment for the growth of tumor tissues, plays an important role in promoting tumor cell proliferation, metastasis, and escaping immune attack (4, 5). Recent studies have shown that copper ions are absorbed through the gastrointestinal tract and exhibit elevated levels in CRC and its microenvironment, mediating its carcinogenesis and development (6). In addition, copper ions can trigger several types of cell death (7, 8). Therefore, understanding the mechanism of action of copper ions in CRC and its targeting effects is crucial for the diagnosis and treatment of CRC.

In general, cells are regulated to maintain a relatively balanced state of copper ion levels through a series of precise manipulations, known as copper homeostasis (9). However, to continuously promote the proliferation and metastasis of CRC cells, they often enrich for copper through high-expression copper ionophores, which is conducive to the proliferation of CRC cells. This leads to a persistently high level of copper ion concentration, making the cells more prone to copper overload events, resulting in copper death (10). Cuproptosis, as a unique mode of cell death, has attracted the attention of medical and pharmaceutical researchers worldwide due to its rapid action (11). Furthermore, it is difficult to develop resistance to copper-based drugs due to the tropism of CRC cells towards copper. Therefore, using copper in the CRC microenvironment to promote the occurrence of copper in colorectal cells is expected to usher in a new era of CRC treatment.

This review article systematically summarizes the role of copper in CRC research and describes its underlying mechanisms. It elaborates on the transport mechanism and biological functions of copper ions, focusing on copper homeostasis, copper growth, and copper death in CRC cells. The article also discusses the unique TME of CRC with high copper and hypoxia, the formation mechanism of this microenvironment, and the promotion of copper death in CRC cells. Additionally, the possible routes and implications for achieving a cuproptosis-based treatment are discussed, along with potential combinations of this new treatment approach with existing treatments.

## 2 The role of copper in CRC

**Figure 1:**
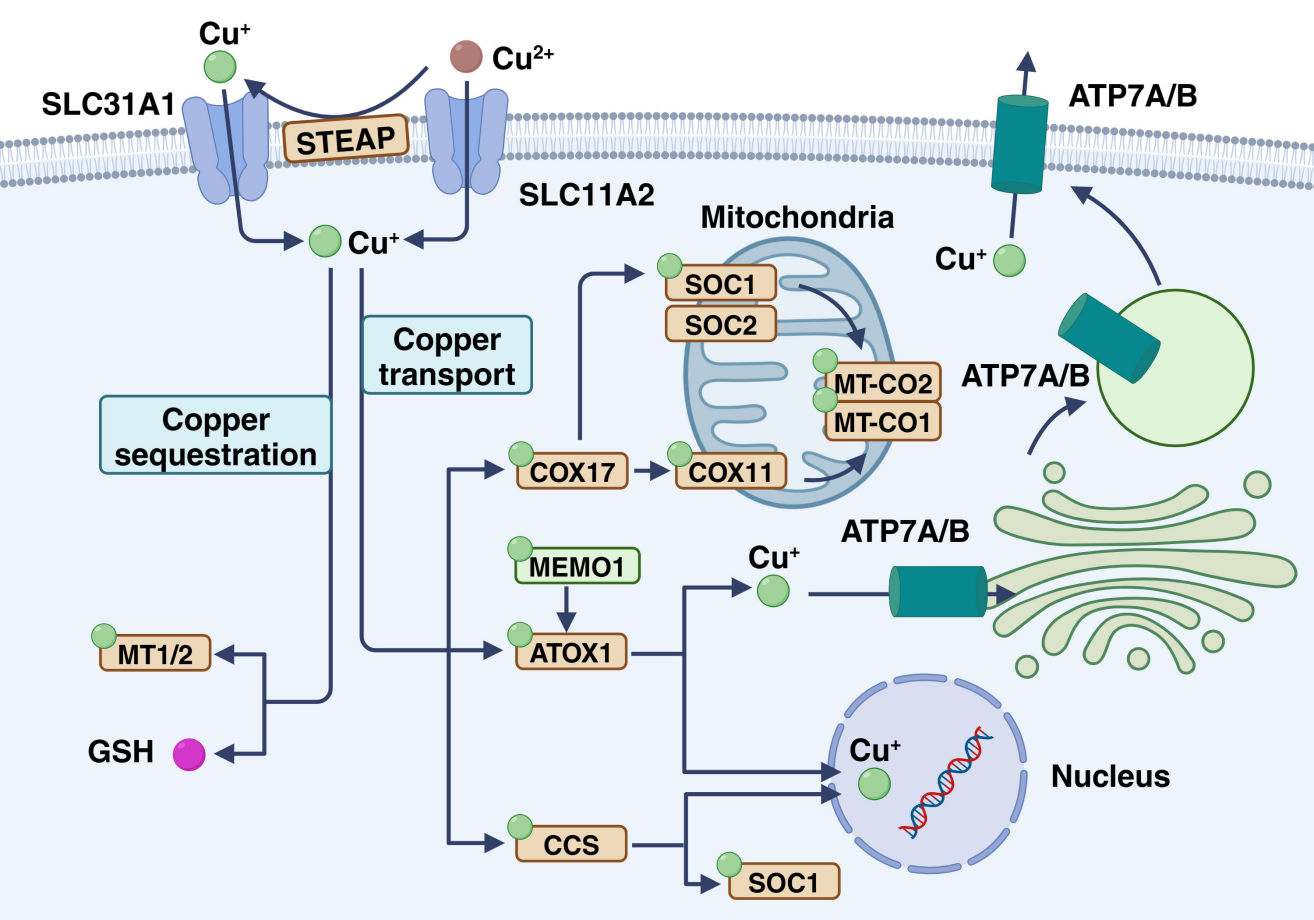
Copper homeostasis in cancer cells. Cu^+^ enters via CTR1 and can follow several pathways: ATOX1 transports copper to ATP7A/B in the Golgi for vesicle storage or export, CCS delivers copper to SOD1, and copper enters mitochondria where COX17, SCO1, SCO2, and COX11 incorporate it into COX for electron transfer. In the plasma, ceruloplasmin carries copper to maintain systemic balance. This figure is created with BioRender (www.BioRender.com).

Copper plays an extremely important role in living cells. It is not only an extremely important trace element that is conducive to cancer metastasis (12), but its ability to accept electrons enables it to convert between monovalent and bivalent, thus delivering electrons. With many complex biochemical reactions (**Figure 1**), copper can bind proteins and low molecular compounds by complexing with histidine, cysteine, and methionine in biological systems, resulting in a range of biochemical effects (13). Numerous studies have shown that elevated levels of copper promote the proliferation and progression of cancer cells in both laboratory and clinical studies (14–16). Notably, the latest research points out that copper acts on a series of proteins associated with cell transduction and death, and its cell physiological and pathological effects are further explained (17). The growth, metastasis, and death of CRC are closely related to copper, which is mainly reflected in copper homeostasis, cuproplasia, and cuproptosis.

### 2.1 Copper absorption, excretion and sequestration in CRC

The uptake and expulsion of copper by CRC cells is a complex process involving multiple mechanisms and proteins. In nature, copper mainly exists as Cu^2+^, but the human body primarily absorbs Cu^+^. Metal reductases like six-transmembrane epithelial antigen of the prostate (STEAP) and duodenal cytochrome b convert bivalent copper to monovalent copper, enhancing its uptake into cells through specific transport proteins at the cell membrane (18, 19). These transporters comprise copper transport proteins (CTRs) and copper ion transport ATPases (ATP7A and ATP7B) (20). After copper enters the cells, a portion is transported through ATP7A and ATP7B, then enriched and stored in the Golgi apparatus and vesicles (21, 22). Copper is primarily excreted from the body through the bile and then eliminated via the intestine (23). At the cellular level, the copper transporter ATP7A/B plays a crucial role in facilitating copper excretion by removing excess copper from the cells. Under normal conditions, these transporters are located in the trans-Golgi network (TGN), where they move copper ions from the cytoplasm into the TGN lumen. With increased copper exposure, ATP7A and ATP7B relocate to either the plasma membrane or the intracellular vesicle compartment. Meanwhile, ATP7A and ATP7B shuttle copper from the trans-Golgi network to the post-Golgi vesicles. These vesicles containing copper can merge with the cell membrane, releasing copper into the surrounding environment (24, 25). Furthermore, in the colorectal tract, copper is reabsorbed through specific transport proteins such as CTR1 to maintain the body’s copper balance.

Under physiological conditions, copper enters the cell membrane and is transported to various cellular substructures, including the Golgi apparatus, cytoplasm, mitochondria, and nucleus (26). The cytoplasm contains high concentrations of glutathione (GSH) and metallothionein (MT), which are natural chelators of copper ions (27, 28). These proteins are more numerous than copper ions, so most of the copper in the cytoplasm is in a chelated state. In addition, chelated copper is not free in the cytosol, but is mediated by ATP7B for vesicle sequestration (29). First, sequestrated copper can maintain a negative concentration gradient of the cytoplasmic membrane, thereby promoting copper uptake by CTR1 along the concentration gradient (30). Second, sequestrated copper can stabilize the concentration of free copper ions, prevent cytotoxicity, and produce reactive oxygen species (ROS) (31, 32). MT and GSH form an intrinsic defense mechanism against copper-induced cytotoxicity. In addition, copper ions can bind to chaperone proteins, specifically the copper chaperone of superoxide dismutase (CCS), which interacts with copper ions and transports them to superoxide dismutase 1 (SOD1), promoting the formation of disulfide bonds, which are essential for their proper structure and enzymatic activity (33, 34). In addition, CCS regulates the distribution of SOD1 in the intermembrane space and cytoplasm in an oxygen-dependent manner. This regulatory mechanism is essential to maintain ROS levels *in vivo* and reduce ROS production by electron transport chains, thereby preventing oxidative damage caused by copper overload (35, 36). Notably, copper sequestration can affect copper concentrations in the tumor microenvironment, thereby affecting the aggressiveness and metastasis of tumor cells (37). The role of copper in angiogenesis and inflammatory response may also be influenced by sequestration, which is the key factor in tumor progression (38, 39).

### 2.2 Copper-driven proliferation and metastasis in CRC

Copper plays a complex and important role in the proliferation of CRC, and this copper-dependent growth phenomenon is known as cuproplasia, which promotes CRC cell proliferation by promoting a series of enzymatic or non-enzymatic reactions in CRC cells (40). Moreover, copper is also closely associated with the spread of CRC (41).

Copper is a cofactor for many enzymes and is involved in key processes such as cellular respiration and antioxidant defense. Excessive accumulation of copper causes oxidative stress that can lead to the degradation of key cancer-suppressor proteins, including p53, affecting cell cycle regulation and inhibiting programmed cell death, thereby improving the viability of CRC cells (42). In addition, Copper can allosterically activate the E2 binding enzyme UBE2D1-UBE2D4, leading to the binding of numerous proteins (including p53) for ubiquitination and degradation (43). Otherwise, copper enzymes activate mammalian kinases such as ULK1 and ULK2, which are downstream targets of the nutrient-sensing kinase mTOR (target of rapamycin) and targets of copper-dependent kinases (44). Notably, disinhibition of ULK1 and ULK2 induces autophagy, allowing CRC cells to recycle intracellular components to support biosynthesis and bioenergy supply, particularly important in the TME (45, 46).

Epithelial-mesenchymal transition (EMT) play a crucial role in the metastasis of CRC, and both processes are closely linked to copper and copper-binding proteins. Copper is essential for the lysyl oxidase (LOX) and lysyl oxidase-like (LOXL) proteins involved in the crosslinking of collagen and elastin. Cancer cells secrete LOX that remodels the extracellular matrix to form pre-metastatic niches, thereby recruiting bone marrow-derived cells that promote EMT in CRC (47, 48). Moreover, the interaction between the copper-mediated hypoxia response element and HIF-1α promotes EMT in CRC through CCS activating the transcription factors ZEB 1, ZEB 2, and Snail (49). Additionally, antioxidant 1 copper chaperone (ATOX1), a copper homeostasis factor, enters the nucleus to activate transcription. This promotes the expression of Cyclin D1 and NADPH oxidase subunit p47phox, critical for cell proliferation and response to oxidative stress. ATOX1 is also a vital copper chaperone protein in human cells that helps maintain copper balance. It carries copper ions from the cytoplasm to the secretory pathway, which is essential for activating copper-dependent enzymes involved in neurotransmitter biosynthesis, iron efflux, neoangiogenesis, wound healing, and blood pressure regulation (50–52). ATOX1 plays a role in regulating the distribution of copper ions within cells, ensuring they are properly allocated to the organelles and molecules that need them. This process helps prevent the improper accumulation and toxicity of copper (53). In addition, ATOX1 protects cells from oxidative stress damage, potentially related to or independent of its copper chaperone role (54). ATOX1 is involved in the regulation and elimination of cellular copper load by promoting the transfer of copper ions to the secretory pathway through its interaction with copper-transporting ATPases such as ATP7A and ATP7B (55). The activity of ATOX1 is affected by the cell’s redox state and GSH balance, as cysteine residues can form disulfide bonds regulated by GSH and glutathione reductase (Grx1) (56). ATOX1 may also be involved in the transfer of copper ions from the copper transporter CTR1 on the cell membrane to intracellular copper chaperone, which is essential for intracellular transport of copper ions (57).

Angiogenesis is crucial for tumor growth and metastasis, with vascular endothelial growth factor regulating vascular growth. Copper ions play a key role in the early stages of tumor vascular formation (38, 58). The activation of angiogenic factors includes basic fibroblast growth factor, vascular endothelial growth factor (VEGF), tumor necrosis factor-alpha, and interleukin-1 (IL-1). These factors, when combined with endothelial cells, promote their transition from G0 to G1 and induce proliferation (59). Moreover, copper ions can increase the production of vasodilator nitric oxide and promote angiogenesis by altering the activity of endothelial nitric oxide synthase (60).

### 2.3 Biological toxicity of copper and cuproptosis in CRC

From the perspective of tumor proliferation and metastasis, CRC seems to maintain the abnormal state of copper metabolism deliberately. CRC maintains high concentrations of copper ions in CRC cells through a series of copper transporters and copper chaperones (20, 53). This is due to the extensive involvement of copper in the maintenance of the microenvironment, angiogenesis, and rapid proliferation in CRC progression (6). This kind of copper enrichment to promote self-proliferation is also called copper proliferation. However, the over-enrichment of copper puts CRC in a very dangerous situation due to the presence of cuproptosis, as excess copper can lead to protein aggregation by binding to the lipid acylated components of the tricarboxylic acid cycle (TCA), which in turn leads to the loss of iron-sulfur protein clusters and ultimately to protein toxic stress and cell death (11).

**Figure 2:**
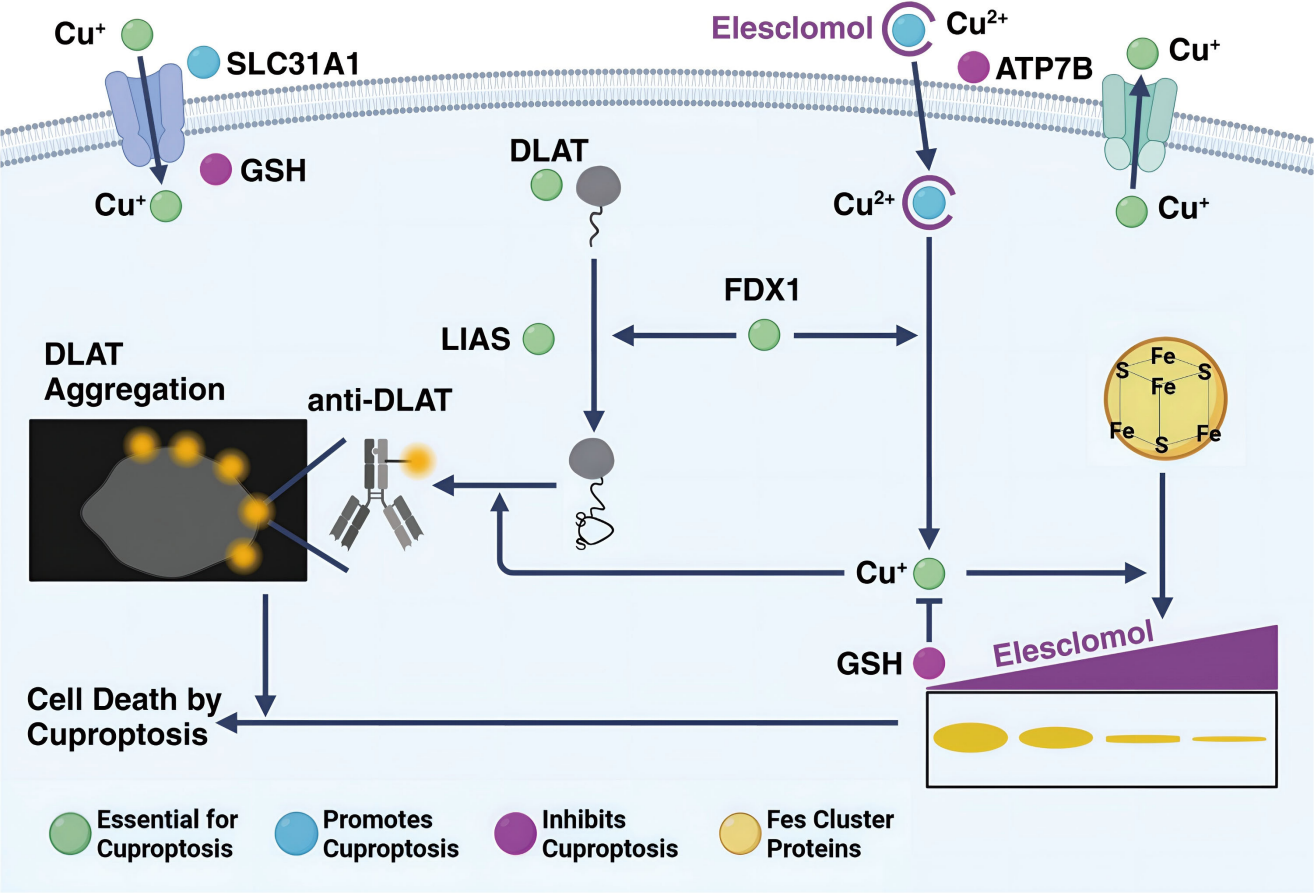
The process of cuproptosis in a cell. Cu^+^ enters the cell via the transporter SLC31A1 and can bind to GSH. ATP7B helps in transporting Cu^+^ into the cell, where it interacts with elesclomol, forming a Cu^2+^-elesclomol complex. This complex can be reduced back to Cu^+^ by FDX1. DLAT, essential for cuproptosis, aggregates when interacting with Cu^+^, and this process is facilitated by LIAS and Fe-S cluster proteins. DLAT aggregation leads to cell death by cuproptosis. The presence of anti-DLAT inhibits DLAT aggregation, thereby preventing cuproptosis. The figure is created with BioRender (www.BioRender.com).

Copper is a heavy metal. Like other heavy metals, copper has biological activity that leads to protein denaturation. However, this does not fully explain the biological toxicity caused by excess copper in cells. Excessive copper-promoting cuproptosis is a complex process. Specifically, Copper enters cells via transporters like SLC31A1/CTR1 and SLC31A2/CTR2 (61), causing copper overload. In that case, copper can enter mitochondria and directly bind to the TCA FDX1-modified TCAT in mitochondrial respiration, to induce the oligomerization of lipoylated dihydro thioamide S-acetyltransferase (DLAT). Oligomerization of lipoylated DLAT causes cytotoxicity and induced cell death. In addition, the reduced ferredoxin 1 (FDX1) reduces the bivalent copper to the monovalent copper, which has greater cytotoxicity and can induce conformational changes in the Fe-S cluster protein, which is more unstable (62). Under the double action, the cells undergo proteotoxic stress, which eventually leads to cell death (**Figure 2**).

Sixteen regulators of cuproptosis were identified, including FDX1, MTF1, DBT, CDKN2A, DLST, DLAT, LIPT1, LIAS, GLS, DLD, PDHA1, PDHB, GCSH, SLC31A1, ATP7A, and ATP7B (11). All of these proteins play important roles in the proliferation and metastasis of CRC cells. At the same time, these known molecules associated with copper death can play an important role in predicting CRC progression. For example, low expression of FDX1 in CRC was associated with a poor prognosis. Analysis of the immune microenvironment showed a significantly lower proportion of CD8+ T cells than in adjacent normal tissues, and the opposite proportion of CD4+ T cells (63).

Non-coding RNAs (ncRNAs) are RNA molecules that do not code for proteins but instead play various regulatory roles in gene expression and cellular processes (64–67). Based on their sizes, tructure, and function, ncRNAs can be classified into different categories based on their size, such as miRNAs, lncRNAs, small siRNAs, and circRNAs. Non-coding RNAs (ncRNAs) related to cuproptosis play crucial roles in the regulation process of CRC cells (**Table 1**). These RNAs include microRNAs (miRNAs) and long non-coding RNAs (lncRNAs), which participate in cuproptosis regulation through different mechanisms and play crucial role in the occurrence of cuproptosis in CRC cells and their prognosis prediction (68–70).

**Table 1:** The comparison of cuproptosis related ncRNA.

Types	Symbol	Location	Protein	References
miRNA	miR34A	1p36.22	ATP7A, ATP7B	(68)
	miR137	1p21.3	ATP7A, ATP7B	(141)
	miR205	1q32.2	ATP7A, ATP7B	(142)
	miR17	13q31.3	ATP7A, ATP7B	(143)
	miR-185	2q11.21	ATP7B	(144)
	miR-98	Xp11.22	ATP7B, PDHB	(145)
	miR-576	4q25	ATP7B, LIPTI	(146)
	miR-664a	1q41	DLAT	(147)
	miR-1271	5q35.2	DLAT	(148)
	miR-3133	2q37.3	DLAT, PDHB	(74)
	miR-452	Xq28	DLAT, LIAS	(149)
	miR-1976	1p36.11	LJAS	(150)
	miR-125b	11q24.1	DLD	(151)
	miR-876	9p21.1	DLD, MTF1	(152)
	miR-125a	19q13.41	DLD, MTF1	(153)
	miR-21	17q23.1	SLC31A1, FDX1	(154)
	miR-708	llq14.1	SLC31A1	(155)
	let-7i	12q14.1	SLC31A1	(156)
	miR-9	1q22	GAS, CDKN2, FDX1	(157)
IncRNA	HOTAIR	12q13.13	CBX2	(158)
	MEG3	14q32.2	ATP7A, ATP7B	(159)
	MATLATI	11q13.1	MTF1	(160, 161)
	LINC02154	Xp22.2	FDX1, DLST	(162)

Most data from EMBL and Genebank. As shown in the table, most of the ncRNA counterparts are ATP7A/B, DLD, DLAT, SLC31A1d, etc., which also points to the direction for future drug opening.

## 3 Tumor microenvironment and CRC sensitivity to cuproptosis

The TME comprises the cellular surroundings of the tumor cells, including immune cells, fibroblasts, endothelial cells, mesenchymal stem cells (MSCs), and extracellular matrix (ECM) (71). Various molecules in the TME maintain CRC cells activity, leading to metastasis, immunosuppression, abnormal angiogenesis, and drug resistance (72, 73). Compared to other types of cancer, the TME in CRC has both common characteristics of a TME and unique high copper levels (74). This unique TME makes CRC more likely to survive in the intestine. However, this microenvironment is characterized by hypoxia and copper-rich, which significantly increases the sensitivity of CRC to cuproptosis (75).

### 3.1 Formation of a hypoxic, high-copper environment

Compared to normal cells, CRC cells have significant differences in biochemical metabolism. Due to the Warburg effect, tumor cells continue to use glycolysis for energy even in the presence of oxygen. This phenomenon is also known as aerobic glycolysis or biochemical reprogramming (76). This effect often leads to the accumulation of lactic acid. The change in biochemical reactions decreases oxygen demand, which further reduces oxygen content in the TME. CRC cells require copper for their growth and spread. These cells often have high levels of copper ionophores, such as ATP7A/B, to absorb and retain copper (77). In addition, the intestine, as the human body absorbs copper, has a higher copper content than other tissues (78). This dual action results in a high concentration of copper in the TME of CRC.

### 3.2 Redox imbalance induced by hypoxia

In the unique biochemical environment surrounding tumor cells, oxygen levels are often low, leading to oxidative stress and disruption of the redox balance in CRC cells (79, 80). Under normal circumstances, cells maintain internal stability through a series of redox reactions to adapt to environmental changes and sustain vital energy processes. However, CRC cells may experience redox imbalance and this imbalance can manifest as increased oxidative stress, where the generation of oxidative species exceeds the capacity of antioxidant defense systems, consequently elevating intracellular oxidative stress levels. Of particular concern is the disruption of the GSH system, which is crucial for the transition of copper valence in CRC cells (81). This heightened oxidative stress can have diverse effects on CRC cells, including increased sensitivity to copper (82, 83). In conditions of oxidative stress, copper’s reactivity may be enhanced, facilitating its interaction with cuproptosis-targeting proteins within the cell, thereby augmenting sensitivity to copper.

### 3.3 Disruption of copper homeostasis in a high-copper environment

Elevated levels of copper in both serum and tissue samples from patients with CRC cell lines indicate a necessity for copper in tumor proliferation. Dysregulation of copper homeostasis and the resultant excess cuproplasia emerge as pivotal factors in CRC development (40, 84, 85). Copper homeostasis is a negative feedback regulation mode that actively regulates the intracellular copper concentration. In general, copper homeostasis is very stable and not easy to destroy, but around CRC cells, due to their copper tropism, the copper content is often an order of magnitude higher than in normal cells. At such high copper concentrations, CRC cells are in fact on the verge of undergoing cuproptosis. Therefore, in an environment with more foreign copper, CRC cells will quickly uptake excessive copper, triggering the process of cuproptosis, and leading to cell death (86). Consequently, in environments abundant with exogenous copper, CRC cells swiftly internalize excessive copper, triggering the process of cuproptosis and eventual cell demise.

## 4 Strategies to enhance cuproptosis in CRC

### 4.1 Therapeutic benefits of cuproptosis in CRC

Cuproptosis is a completely new mode of cell death, and treatment with other inhibitors of known cell death mechanisms — including ferroptosis, necroptosis, and oxidative stress (87–89) — has failed to eliminate copper ionophore-induced cell death. Due to drug resistance, current treatments like chemotherapy, radiotherapy, immunotherapy, and targeted therapy often do not effectively combat colorectal cancer (2). However, a cuproptosis-based treatment could effectively prevent this. First, the cause of cuproptosis is an overload of copper ions in cells. However, CRC cells require high levels of copper to maintain their proliferation and metastasis, making it difficult to eliminate this cause. Secondly, the sites of cuproptosis, such as FDX1 and DLAT, are relatively conserved. This suggests that CRC cells may struggle to evade cuproptosis through mutations (11). The cuproptosis pathway offers advantages such as a brief duration, rapid progression, widespread occurrence, and easy pharmaceutical targeting, demonstrating its potential for treating colorectal cancer.

### 4.2 Promotion of cuproptosis via GAPDH inhibition

**Figure 3:**
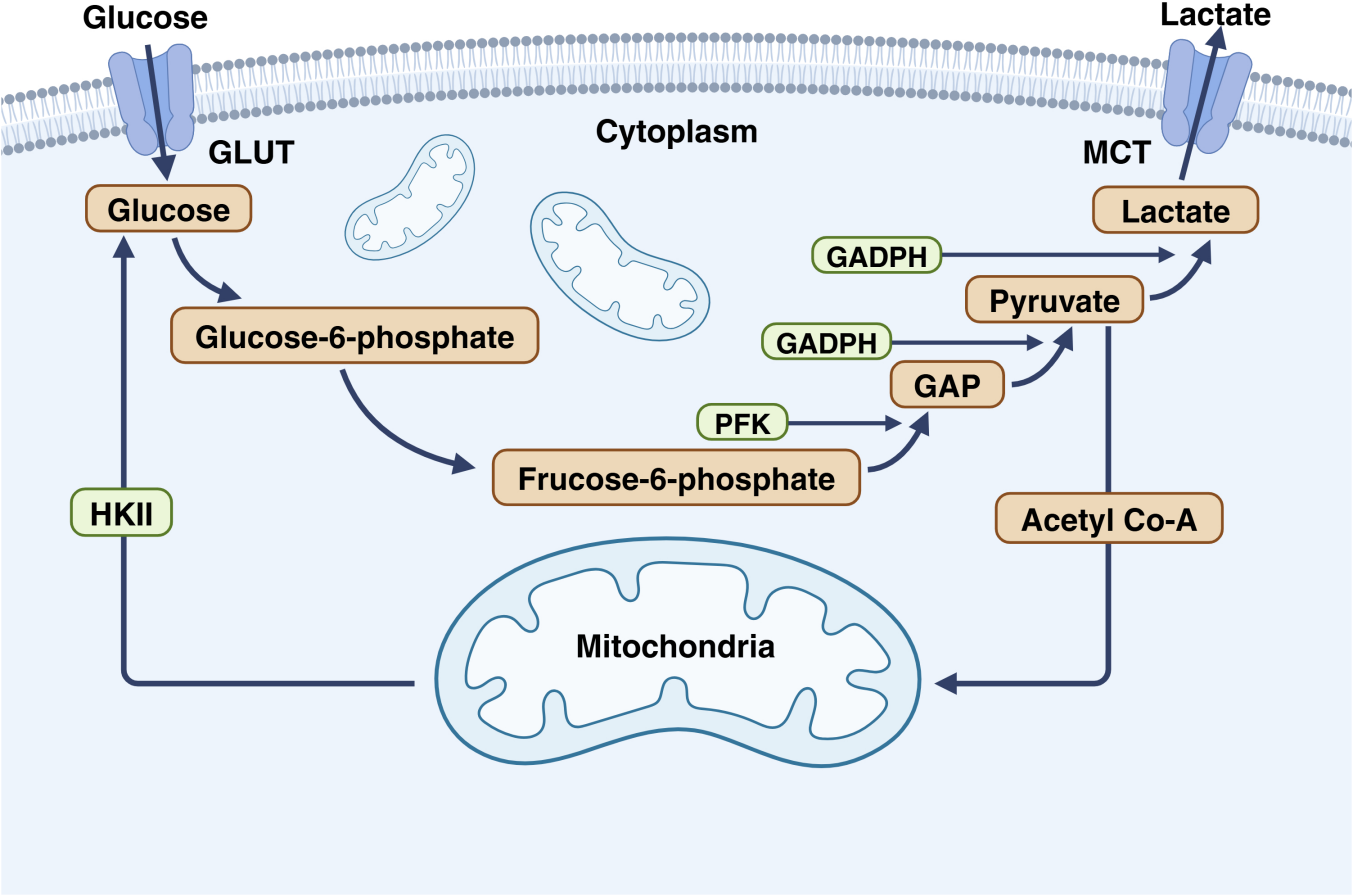
The important role of GAPDH in glycolysis. In glycolysis, glucose enters cells via GLUT and is converted to 6-phosphogluconate by hexokinase. Phosphoglucose isomerase then converts it to fructose-6-phosphate, which is transformed into GAP by PFK. For glycolysis products to enter the TCA cycle in the mitochondria, GAP must be converted to pyruvate by GAPDH and then to acetyl-CoA. This underscores the importance of GAPDH in converting glucose to acetyl-CoA. This figure is created with BioRender (www.BioRender.com).

Glyceraldehyde-3-phosphate dehydrogena (GAPDH) is a key enzyme in the sixth step of glycolysis, catalyzing the conversion of glyceraldehyde-3-phosphate to 1,3-bisphosphoglycerate (90). In the process of glycolysis, glucose enters cells via GLUT and is converted to 6-phosphogluconate under the action of hexokinase (91). Subsequently, it is isomerized to fructose-6-phosphate by phosphoglucose isomerase. Then, glyceraldehyde-3-phosphate (GAP) is formed under the catalysis of phosphofructokinase (PFK) (92). However, for glycolysis products to enter the mitochondria and participate in the TCA cycle, they must be converted to pyruvate by GAPDH before further conversion to acetyl-CoA to enter the mitochondria. This highlights the significance of GAPDH in converting glucose into the universal reactant acetyl-CoA in cells (**Figure 3**). Numerous studies have confirmed that it is a potential therapeutic approach to inhibit aerobic glycolysis in tumor cells (93–95).

GAPDH is an important enzyme in aerobic glycolysis. If the activity of GAPDH is inhibited, it will directly inhibit the whole aerobic glycolysis, making the energy produced by yeast greatly reduced (96–98). In CRC, due to their special biochemical environment (99, 100), tumor cell cells mainly rely on glycolysis for energy, so inhibiting the activity of GAPDH will directly lead to the reduction in energy gain leading to CRC. In the case of energy shortage, the metabolic speed in CRC is weakened, and the copper in cells will not be excluded by ATP7A/B, which will cause copper retention in cells, leading to the destruction of copper homeostasis, and eventually excessive copper accumulation and cuproptosis. In addition, the inhibition of GAPDH will also reduce acetyl-CoA in CRC. In such cells, both protein synthesis and DNA replication will slow down, leading to the decreased proliferation, diffusion and stress resistance of CRC. When cuproptosis occurs in CRC, cells will be unable to remove the lipoylated proteins that are aggregation and allowing them to exert toxic effects. Overall, the CRC showed an increased sensitivity to cuproptosis.

In fact, under normal circumstances, tumor cells produce energy through glycolysis, while mitochondrial respiration is strictly inhibite (101). However, this does not mean that tumor cells cannot undergo mitochondrial respiration. In the case of GAPDH inhibition, CRC compensates for the lack of ATP and acetyl-CoA by engaging in aerobic metabolism such as aerobic respiration and lipid metabolism (102). Under this circumstance, due to the opening of aerobic respiration, increased permeability of the mitochondrial membrane, and changes in the redox environment in cells, Cu (II) ions are more easily reduced to Cu (I) ions and enter mitochondria to trigger cuproptosis.

### 4.3 Promotion of cuproptosis via copper ion carriers

In the first step of cuproptosis, the copper ionophore mediate the entry of copper ions into cells, playing an extremely important role in the development of cell cuproptosis. Among them, SLC31A1, responsible for transporting Cu (I) ions, plays a pivotal role (61). On the surface of tumor cells, Cu (II) ions are reduced to Cu (I) ions by STEAP, after which SLC31A1 facilitates their entry into the cell, triggering subsequent reactions (103). Experimental evidence has demonstrated that the use of CTR inhibitors can significantly alleviate the extent of cuproptosis, underscoring the regulatory role of CTR in cuproptosis (104).

### 4.4 Promotion of cuproptosis via mitochondrial respiration

**Figure 4:**
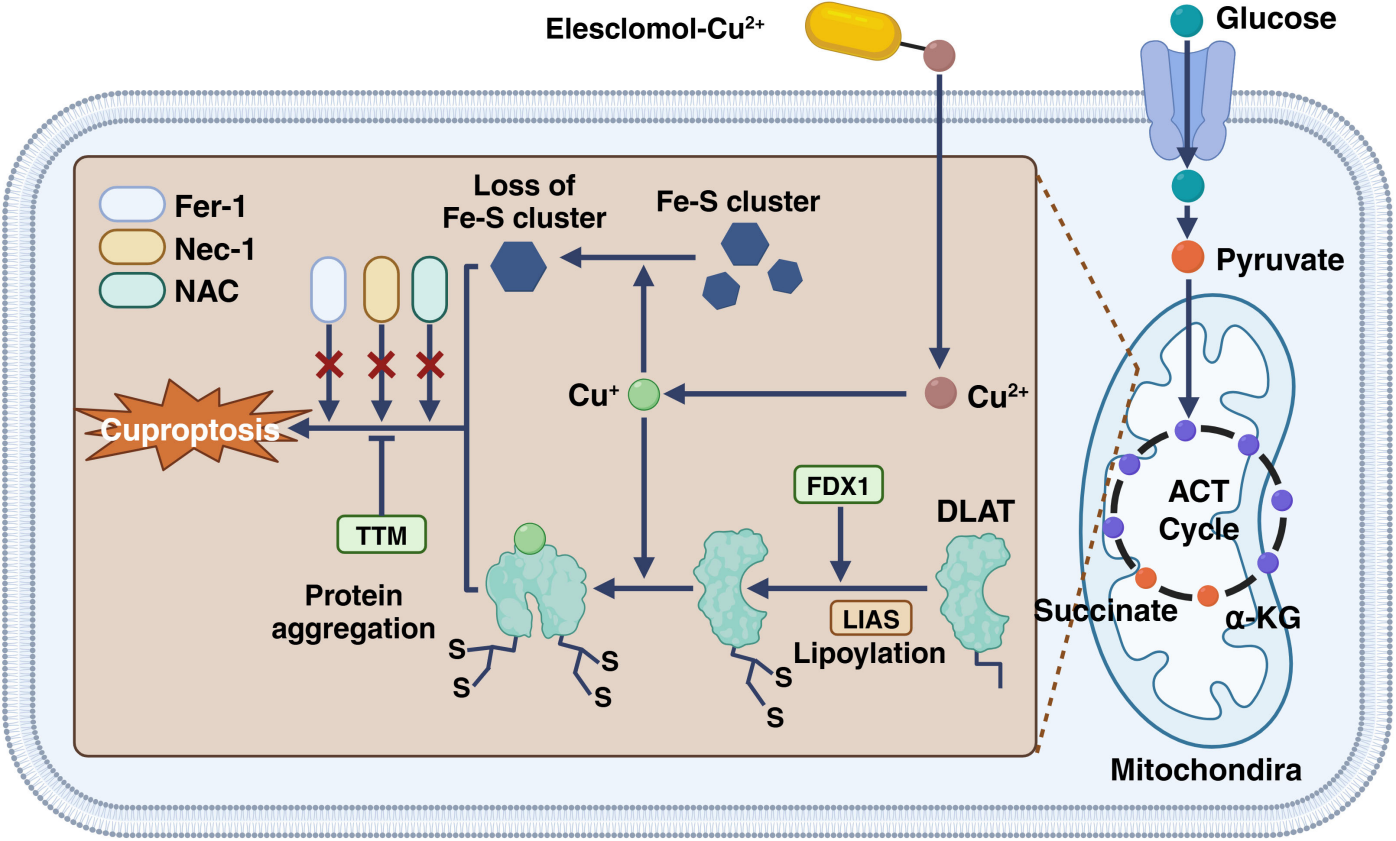
The inhibition of electron transport chain complexes I and II significantly reduces cuproptosis. Copper ions modify several metabolic enzymes through lipoylation, a conserved posttranslational modification. Although few proteins are lipidated in mammalian cells, all lipidated proteins are involved in the TCA cycle. One such lipidated protein, DLAT, is part of the pyruvate dehydrogenase complex. Copper can bind directly to DLAT, promoting disulfide bond-dependent aggregation. This figure is created with BioRender (www.BioRender.com).

Mitochondria are the main targets of cuproptosis, characterized by oxidatively damaging mitochondrial membranes and impaired enzyme function in the TCA cycle (105–107). Metabolomic analysis of cells treated with copper ions reveals a time-dependent increase in the dysregulation of several TCA cycle-related metabolites. Additionally, significant attenuation of cuproptosis is observed upon inhibition of electron transport chain complexes I and II. Furthermore, copper ions alter a range of metabolic enzymes by lipoylation (11), a highly conserved posttranslational modification, and all lipidated proteins participate in the TCA cycle, although relatively few proteins are lipidated in mammalian cells. One such lipidated protein is DLAT, a subunit of the pyruvate dehydrogenase complex, and copper can directly bind to DLAT to promote the disulfide bond-dependent aggregation of lipidated DLAT (108, 109) (**Figure 4**). When tumor cells are in hypoxia, mitochondrial respiration is inhibited, and copper is difficult to enter mitochondria, and cuproptosis is greatly inhibited.

### 4.5 Promotion of cuproptosis via FDX1 and protein lipidation

FDX1 is a very small iron-sulfur protein that transfers electrons from NADPH to mitochondrial cytochrome P450 by ferredoxin reductase, involved in the aerobic metabolism of steroids, vitamin D, and bile acids (110, 111). It can not only play the role of electron transport but also can reduce the Cu (II) to the more toxic form (112, 113). The researchers found that if the FDX1 gene is knocked down with CRISPR-Cas9, not only will mitochondrial respiration be inhibited, but copper will be more difficult to enter mitochondria, but copper will also weaken its toxicity due to its inability, thus greatly alleviating the effect of cuproptosis on cells (114).

In addition, protein lipoylation also plays a key role in the cuproptosis process (9, 11, 115, 116). Lipoylation is not common in the biochemical environment of the human body. The proteins that can undergo lipoylation are concentrated in mitochondrial respiration, mainly TCA (117), among which the main roles are LIPT1, DLD, LIAS, DLAT, PDHA1, and PDHB (118, 119). In the cuproptosis process, TCA related proteins are lipoylated and aggregated, resulting in the stalling of whole mitochondrial respiration and great biotoxicity (120). Knockout of the above protein genes can rescue the cell toxicity of copper ions.

It should be noted that FDX1 is an upstream regulator of protein lipoylation throughout, and in fact that lipoylation of proteins is inaccessible in the absence of FDX1. FDX1, as an important component of the electron transport chain, does not work without mitochondrial respiration (111, 121), which also shows that the necessary condition for cuproptosis is mitochondrial respiration.

## 5 Integrating cuproptosis therapy with existing CRC treatments

### 5.1 The combination between cuproptosis and immunologic therapy

Immunotherapy of PD-1 with PD-L1 is an immunotherapy strategy used to enhance the aggression of the immune system against cancer. Programmed cell death protein 1(PD-1) is a cell-surface receptor, whereas programmed death-ligand 1(PD-L1) is its ligand (122, 123). Under normal circumstances, the binding of PD-1 to PD-L1 can inhibit the activity of T cells, thus preventing the excessive activation of the immune system and causing its own tissue damage (124). However, in some cancers, tumor cells or other cells will overexpress PD-L1 to inhibit the surrounding T cells by binding to PD-1 to evade immune surveillance (125). The rationale of PD-1 and PD-L1 immunotherapy is to use antibodies against anti-PD-1 or anti-PD-L1 to block the binding between PD-1 and PD-L1, thus releasing the inhibitory effect on T cells and enhancing the tumor attack of the immune system (122, 123). This therapeutic strategy has been shown to be effective in a variety of cancers, including melanoma, non-small cell lung cancer, CRC, etc. However, in the actual treatment, the effect of PD-1 and PD-L1 immunotherapy is not effective, because the tumor cells will continuously make PD-1 to the cell surface.

For PD-1/PD-L1 immunotherapy to continue to work, it needs to inhibit tumor cells to continuously make PD-1. This effect can be achieved by drugs that induce cuproptosis, which reduces PD-1 expression by promoting tumor cell death or interfering with material synthesis in the cells. In addition, the strong specificity between PD-1 and PD-L1 makes it possible to make relevant targeted drugs, which will greatly improve the anticancer properties of drugs and reduce the damage to normal cells in the human body. In the latest study, researchers developed a sodium alginate hydrogel composed of sodium dichloroacetate copper and galactose to induce sustained atrophy, resulting in a reduction of PD-L1 on the surface of CRC cells. When implanted into the tumor, the preformed hydrogel can further crosslink in the presence of physiological calcium ions (Ca^2+^), forming a hydrogel that controls the release of elesclomol-Cu^2+^ (ES-Cu) and galactose. This hydrogel effectively induces DLAT oligomerization and copper-induced cell death in CRC cells. Additionally, the radiation-induced upregulation of PD-L1 is abrogated in the presence of the hydrogel, which releases ES-Cu and galactose. Consequently, the tumor’s sensitivity to radiotherapy and immunotherapy is significantly enhanced, further prolonging the survival of tumor-bearing mice with both local and metastatic tumors (126).

### 5.2 The combination between cuproptosis and chemotherapy

Chemotherapy is the cornerstone of treatment for CRC. After years of development, several effective drugs, including capecitabine, 5-fluorouracil, and oxaliplatin, are widely used in clinical practice (127). In many clinical treatments, drug resistance of CRC often occurs, leading to treatment failure (128). This is due to the unique microenvironment and metabolic pattern of CRC. Treatment targeting cuproptosis can avoid and even utilize the microenvironment characteristics of CRC. While conventional chemotherapeutic agents typically work by blocking or interfering with DNA replication, cuproptosis operates through a different mechanism, that is, excess copper can lead to protein aggregation by binding to the lipid-acylated components of the TCA, which in turn leads to the loss of iron-sulfur protein clusters and ultimately leads to protein toxic stress and cell death (129). Additionally, there is a type of drug that can enhance cuproptosis by targeting GAPDH, and these drugs can block glycolysis, reduce the energy and raw material source needed for DNA replication, and inhibit the proliferation of CRC cells (130). Based on this, the combination of traditional chemotherapy and targeted cuproptosis drugs will greatly improve the effect of traditional therapy.

## 6 Discussion

Overall, copper ions play an important role in the development of CRC. Copper is an essential trace element inside cells and is involved in various biochemical processes, but its abnormal accumulation in CRC is closely related to the proliferation and metastasis of tumor cells. Copper ions affect cell signaling and apoptosis, which in turn promote tumor proliferation and metastasis. In the TME of CRC, the high levels of copper ions interact with the metabolic properties of tumor cells to create a hypoxic, high-copper environment. This environment not only promotes the survival of tumor cells but also increases their sensitivity to cuproptosis. Studies have shown that the induction of cuproptosis in CRC cells can be achieved by regulating the level of copper ions, offering new insights for CRC treatment.

The mechanism of cuproptosis is very different from that of ferroptosis and apoptosis (11). Compared with apoptosis, cupoptosis is more like a stress response to high concentrations of copper ions. It does not have a large number of complex genes and proteins behind apoptosis, nor does it have the characteristic apoptotic bodies and keep cell membranes intact like apoptosis (131). Cuproptosis is more efficient and has a shorter pathway than ferroptosis. In CRC, cuproptosis is more likely to occur because of copper accumulation, which is necessary for the tumor’s progression (132). However, cuproptosis, ferroptosis, and apoptosis are not unrelated. Previous studies have shown that disorders of copper metabolism can lead to cancer cell death through apoptosis, paraptosis, ferroptosis, and caspase-independent cell death (133–136). In addition, both copper and ferroptosis lead to increased intracellular oxidation levels, especially the disruption of the GSH redox system, and the imbalance of intracellular redox can lead to apoptosis (137). This shows that there is a certain correlation and similarity between the three in terms of progress.

Treatment regimens based on cuproptosis present several advantages for CRC. Due to natural copper accumulation in the TME, these treatments require less intervention to improve efficiency compared to other drug regimens. Cuproptosis also features a shorter death pathway, making it harder for CRC to develop drug resistance. Additionally, the TME has the highest copper concentration in the body, allowing for targeted treatment that minimizes systemic toxicity and side effects. These factors highlight the significant potential of using cuproptosis to treat CRC. However, cuproptosis-based treatment regimens have some limitations, as a critical step in cuproptosis is the entry of copper into the mitochondria to bind to the lipoylated protein in TCA, which is important for mitochondrial opening, or mitochondrial respiration in the cell (138). Tumor cells primarily use glycolysis for energy due to the Warburg effect, which keeps their mitochondria nearly closed and limits copper entry, posing a challenge for cuproptosis-based treatments (139). However, this is not an insurmountable issue, and combining glycolysis-inhibiting drugs with copper proptosis could effectively address this problem, as the aerobic oxidation of tumor cells will compensate for the increase (140). In the future, the combination of related drugs will provide a broader prospect for the cuproptosis-based treatment.

Overall, studying how copper ions affect immune cells, stromal cells, and other components in the TME, as well as how these cells in turn affect the metabolism of copper ions, will enhance our comprehensive understanding of the TME, copper, and CRC. In addition, developing drugs that can induce cuproptosis in CRC cells, particularly targeting key aspects of copper metabolism and cuproptosis, as well as exploring the potential of combining cuproptosis with traditional chemotherapy and immunotherapy. This will further innovate and improve the mode of CRC treatment and enhance the quality of treatment and living standards for patients. The cuproptosis-based drug is showing its great potential in treating CRC and will pioneer a new era of CRC therapy.
